# Clinicopathological characteristics and outcome predictors of anti-glomerular basement membrane glomerulonephritis

**DOI:** 10.1080/0886022X.2022.2147673

**Published:** 2022-11-21

**Authors:** Yifei Ge, Kang Liu, Guang Yang, Xiangbao Yu, Bin Sun, Bo Zhang, Yanggang Yuan, Ming Zeng, Ningning Wang, Changying Xing, Huijuan Mao

**Affiliations:** Department of Nephrology, The First Affiliated Hospital of Nanjing Medical University, Nanjing, China

**Keywords:** Anti-glomerular basement membrane disease, glomerulonephritis, renal pathology, risk factors, renal survival, patient survival

## Abstract

**Objective:**

To explore the clinicopathological features of anti-glomerular basement membrane (anti-GBM) glomerulonephritis (anti-GBM-GN) and the prognostic values of clinical and laboratory indicators at diagnosis on renal and patient survival.

**Methods:**

A total of 76 patients (34 males and 42 females) with anti-GBM-GN who were hospitalized in the First Affiliated Hospital of Nanjing Medical University between January 2010 and June 2021 were included in this study. The baseline clinical features, histopathological data from renal biopsies, and predictors of renal and patient survival were retrospectively analyzed.

**Results:**

Among the 76 patients, the median serum creatinine at diagnosis was 618.0 (350.98, 888.25) μmol/L and the median estimated glomerular filtration rate (eGFR) was 6.62 (4.39, 14.41) mL/min. Of these 76 patients, 55 (72.4%) received initial kidney replacement therapy (KRT) and 39 (51.3%) received plasma exchange or double-filtered plasmapheresis (DFPP). During a median follow-up duration of 28.5 (6.0, 71.8) months, 53 (69.7%) patients progressed to kidney failure with replacement therapy (KFRT) and received maintenance dialysis. Initial KRT (HR = 3.48, 95% CI = 1.22–9.97, *p* = 0.020) was a significant risk factor for renal survival. During the follow-up, 49 (64.5%) of 76 patients survived. Age (≥60 years, HR = 4.13, 95% CI = 1.65–10.38, *p* = 0.003) and initial KRT (HR = 2.87, 95% CI = 1.01–8.14, *p* = 0.047) were predictive of patient survival.

**Conclusions:**

Among patients with anti-GBM-GN, initial KRT at presentation was predictive of KFRT while older age and initial KRT were associated with higher all-cause mortality.

## Introduction

Anti-glomerular basement membrane (anti-GBM) disease is a rapidly progressive and relatively rare autoimmune disease. It is estimated that there are only 1–2 cases per million population per year [1,2]. The disease mostly affects glomerular capillaries (usually leading to rapidly progressive glomerulonephritis) and pulmonary capillaries, which may be characterized by alveolar hemorrhage. This condition is often referred to as Goodpasture’s syndrome. Renal involvement is the predominant clinical presentation in most of these patients, and histopathological features are glomerular fibrinoid necrosis and crescent formation [3]. The term ‘anti-GBM glomerulonephritis (anti-GBM-GN)’ was refers specifically to renal involvement in anti-GBM disease. The tissue injury of anti-GBM disease is directly mediated by pathogenic anti-GBM antibodies, which specifically binds to type IV collagen α3 chain of the basement membranes of kidney, lung, and brain choroid plexus [4,5]. Anti-GBM antibodies in the serum of most patients are positive, which can be the key diagnostic basis for the disease. In addition to the positive anti-GBM antibodies, about one-third of the patients are also concomitantly positive for anti-neutrophil cytoplasmic antibody (ANCA), mainly myeloperoxidase (MPO)-ANCA positive [6,7]. The main treatment of anti-GBM disease is to remove pathogenic autoantibodies by plasma exchange in the early stage, and then use corticosteroids and cytotoxic drugs to eliminate the continuous production of autoantibodies and their damage to tissue [8]. However, immunosuppressive programs are highly heterogeneous among different populations. Regarding the research on anti-GBM disease, so far, there has not been any randomized study to determine the best treatment option. The standard treatment can make most patients anti-GBM autoantibodies serologically disappear. In addition to the above-mentioned classic treatment regimens, anti-CD20 rituximab is recommended for patients with refractory or relapsed anti-GBM disease, as well as patients with CYC intolerance [9]. In the past decades, these therapies, together with early diagnosis and better supportive care, have significantly improved the poor prognosis of patients with anti-GBM diseases [10–12]. Although prognosis has improved, the rapid deterioration of the disease still leads to rapid progression to kidney failure with replacement therapy (KFRT), and about 55% of patients requires dialysis at the time of diagnosis [10]. Some studies have confirmed that older age, oliguria, dialysis dependency at presentation, percentage of glomerular crescents, and extensive interstitial inflammatory cell infiltration were important factors affecting renal prognosis. However, other researchers reported that the only predictor of renal prognosis was serum creatinine at presentation [12–16]. A number of reports indicate that the two main causes of death from anti-GBM disease are still refractory pulmonary hemorrhage related to severe acute respiratory distress syndrome, or severe bacterial infection related to immunosuppression [14,15,17]. The advanced age, hypertension, number of plasma exchanges, and oliguria at diagnosis as well as the serum level of anti-GBM antibodies were predictive of poor patient outcome [12,13,15,16]. However, there are relatively few studies on renal survival and long-term prognosis in patients with anti-GBM-GN, and the number of patients is small. Therefore, the purpose of this retrospective study was to evaluate the clinical and laboratory data of patients with anti-GBM-GN to determine the risk factors affecting prognosis.

## Methods

### Ethics statement

The study protocol was approved by the Ethics Committee of the First Affiliated Hospital of Nanjing Medical University (Nanjing, China) (Ethical Approval No.: 2018-SR-180). Written informed consent was obtained from all of the patients.

### Study design and patients

In this retrospective cohort study, 76 anti-GBM disease patients newly diagnosed were enrolled from January 2010 to June 2021 at the First Affiliated Hospital of Nanjing Medical University. The inclusion criteria of patients were that the clinical manifestations were consistent with anti-GBM-GN, and there were positive circulating anti-GBM antibodies detected by ELISA or immunofluorescence and/or linear IgG fluorescence along the GBM on renal biopsy. Patients were also included in the study if they were double positive for anti-GBM antibodies and ANCA, but were excluded if they had any other renal disease at same time. Cases with more missing data were also excluded. The study cohort was followed up from January 2010 to December 2021.

### Clinical and laboratory parameters

Baseline medical information included demographics, medical history, baseline laboratory tests including hemoglobin, serum albumin, urea, creatinine, C-reactive protein, rheumatoid factor, etc. were recorded. Anti-GBM antibodies and ANCA levels were measured using enzyme-linked immunosorbent assay (ELISA) kit (EUROIMMUN, Lübeck, Germany). The estimated glomerular filtration rate (eGFR) was calculated using the Chronic Kidney Disease Epidemiology Collaboration (CKD-EPI) equation. Oliguria was defined as urinary output of <400 mL/24 h while anuria was defined as urinary output of <100 mL/24 h.

### Renal biopsy pathology

Of the 76 patients, 38 (50.0%) underwent renal biopsy at diagnosis or before the initiation of immunosuppressive therapy. Renal tissue specimens were subjected to light microscopy, immunofluorescence, and electron microscopy according to standard procedures. As reported by Berden et al., we classified the renal histopathology of anti-GBM-GN according to ANCA-GN into four types: focal (>50% normal glomeruli), crescentic (>50% of glomeruli with cellular crescents), sclerotic (>50% of glomeruli with global sclerosis), or mixed (no predominant glomerular phenotype) [18]. According to the degree of involvement of tubulointerstitial lesions, the degree of interstitial fibrosis and tubular atrophy was divided into mild (<25%), moderate (25–50%), and severe (>50%). Similarly, interstitial inflammation was also divided into mild (<25%), moderate (25–50%), and severe (>50%). All renal biopsy histopathological sections were independently read and reviewed by two experienced renal pathologists. If there were differences, they resolved through discussion and consensus.

### Treatment

The patient received prednisone (0.5–1 mg/kg/day) for 6–8 weeks, followed by tapering of prednisone. The most commonly used immunosuppressive regimen was intravenous cyclophosphamide at 0.75–1.0 g/m^2^ body surface area twice monthly. MMF was also used in a very small number of patients. In severe cases, such as pulmonary hemorrhage or renal histopathology indicating crescentic nephritis, the patients received intravenous methylprednisolone (500 mg/d) pulse therapy for three consecutive days, as well as plasma exchange, double-filtered plasmapheresis (DFPP) or immunosorbent therapy.

### Follow-up

Patients were followed-up until death, progression to KFRT, or until 31 December 2021. KFRT was defined as eGFR <15 mL/min/1.73 m^2^ or receiving maintenance dialysis therapy for more than 3 months.

### Statistical analyses

We tested if continuous variables had a normal distribution using the Kolmogorov–Smirnov test. Continuous variables are represented as the mean ± standard deviation or median with interquartile range if the distribution of variables was skewed. The unpaired Student’s *t*-test or Mann–Whitney’s *U*-test was performed for comparison between groups. In the case of dichotomous variables, group differences were examined by Pearson’s Chi-square or Fisher’s exact test as appropriate. Overall survival and renal survival were estimated by the Kaplan–Meier method. Variables related to survival were assessed using the log-rank test in a univariate analysis, and those potential risk factors with probability (*p*) value <0.1 were further included in a multivariate analysis using a Cox proportional hazards model after disregarding the effect of multicollinearity. The multicollinearity among included variables was evaluated by Spearman’s correlation analysis, while the absolute value of the correlation coefficient >0.7 was considered to be significant. In addition, some model covariates were selected on the basis of clinical judgment and were retained in the model regardless of statistical significance. The cutoff values of continuous variables were dependent upon the lower or upper limits of the reference range, the median values or the experience from previous literatures. The results of regression analysis are expressed as the hazard ratios (HRs) and 95% confidence intervals (CIs). All analyses were performed using IBM SPSS Statistics for Windows, version 20 (IBM Corporation, Armonk, NY). Statistical tests were two sided and *p* values <0.05 were considered statistically significant.

## Results

### Demographic and clinical data

The baseline demographics, clinical characteristics of the 76 patients included in this study are shown in Table 1. The cohort consisted of 34 males and 42 females. The median age, serum creatinine level at diagnosis, and eGFR (CKD-EPI) were 60.0 (44.5, 68.0) years, 618.0 (350.98, 888.25) μmol/L, and 6.62 (4.39, 14.41) mL/min, respectively. Among all 76 patients, 29 (38.2%) cases showed gross hematuria, 21 (27.6%) cases showed oliguria or anuria, and 15 (19.7%) cases were combined with MPO-ANCA positive.

**Table 1.** Baseline data of 76 patients with anti-GBM disease.

Variables	Value
Male (*n*, %)	34 (55.3%)
Age (years)	60.0 (44.5, 68.0)
Duration of renal disease (months)	1.0 (0.5, 2.0)
Oliguria/anuria (*n*, %)	21 (27.6%)
Macroscopic hematuria (*n*, %)	29 (38.2%)
Initial KRT (*n*, %)	55.0 (72.4%)
MPO-ANCA positivity (*n*, %)	15.0 (19.7%)
Serum creatinine (μmol/L)	618.0 (350.98, 888.25)
eGFR (mL/min/1.73 m^2^)	6.62 (4.39, 14.41)
CRP (mg/L)	30.00 (3.42, 92.15)
RF (IU/mL)	10.40 (9.69, 11.35)
Serum albumin (g/L)	28.00 (23.95, 31.73)
Hemoglobin (g/L)	84.50 (73.75, 97.00)
Urine red blood cell (/μL)	1362.10 (335.85, 2641.15)
Urine protein (g/24 h)	1.27 (0.59, 3.96)
Lung involvement (*n*, %)	22 (28.9%)
Treatment (*n*, %)	
Plasma exchange or DFPP (*n*, %)	39 (51.3%)
GC + CTX	62 (81.6%)
GC alone	7 (9.2%)
No immunosuppressive therapy	5 (6.6%)

MPO-ANCA: myeloperoxidase-anti-neutrophil cytoplasmic antibody; eGFR: estimated glomerular filtration rate; KRT: kidney replacement therapy; CRP: C-reactive protein; RFs: rheumatoid factors; DFPP: double-filtration plasmapheresis; GC: glucocorticoid; CTX: cyclophosphamide.

Oliguria was defined as urinary output of <400 mL/24 h while anuria was defined as urinary output of <100 mL/24 h. The Hospital Reference Laboratory normal range for CRP was 0–8 mg/L; RF was 0–20 IU/mL; and urine red blood cell was 0–17/μL.

In terms of treatment, 39 of 76 patients received plasma exchange, DFPP, or immunoadsorption therapy. All these 39 patients were treated with glucocorticoids (GCs) plus intravenous CTX. The most common reason for deciding not to use of plasma exchange was the vast majority of glomerulosclerosis, or a small number of mild cases with slightly elevated anti-GBM titer and normal renal function. Among the 71 patients who received prednisone induction therapy, 38 individuals received methylprednisolone intravenous pulse therapy. A total of 62 (81.6%) patients received GCs plus intravenous CTX, and seven (9.2%) received GCs alone. Two patients received other immunosuppressive agents (mycophenolate mofetil, *n* = 2).

### Clinical and renal histopathologic characteristics

Of the 76 patients, 38 underwent renal biopsy. Renal biopsy was not performed in some patients with very advanced age or KFRT at presentation who had contraindications to renal biopsy. Among the 38 patients who underwent renal biopsy, six (15.8%) were classified as focal type, 22 (57.9%) as crescentic, five (13.2%) as mixed and five (13.2%) as sclerotic. There was no significant difference in clinical characteristics such as age, serum creatinine, hemoglobin, and serum albumin among the four groups. The score of acute tubulointerstitial lesions in focal group was significantly lower than that in crescentic and sclerotic group (*p* < 0.05). In terms of tubular atrophy and interstitial fibrosis, the score of sclerotic group was significantly higher than that of focal and crescentic group (*p* < 0.05). As expected, the percentage of normal glomeruli in focal group was significantly higher than that in crescentic and sclerotic group (*p* < 0.05) (Supplementary Table 1).

### Clinical characteristics of anti-GBM-GN patients by MPO-ANCA positivity

Among the 76 patients, 15 were accompanied by MPO-ANCA positive. Patients with MPO-ANCA positive were approximately 10 years older (*p* = 0.030) than those without MPO-ANCA positive. Of these 15 patients, five underwent renal biopsy. The immunofluorescence of these five patients mainly showed that IgG deposited linearly along GBM. Electron microscopy showed that there was no electronic dense deposit. Among the 15 MPO-ANCA positive patients, 13 (86.7%) showed acute kidney injury and received kidney replacement therapy (KRT) at presentation, while the percentage was 68.9% (42/61) in patients without MPO-ANCA positive. In terms of biochemical parameters and renal pathological features, there was no significant difference between patients with and without MPO-ANCA positive (Supplementary Table 2).

### Predictors of renal survival in anti-GBM-GN patients

Among the 55 patients who received initial KRT, eight (14.5%) had improved renal function after treatment and discontinued KRT, while 47 (85.5%) continued on maintenance KRT. During a median follow-up of 1 (1.0, 5.0) months, six of 21 patients who did not receive initial KRT progressed to KFRT and received maintenance KRT. The level of anti-GBM antibody in dialysis-dependent group was significantly higher than that in non-dialysis-dependent group (158.5 (115.5, 198.9) vs. 50.1 (34.6, 112.7), *p* < 0.001). The initial renal function status (initial dialysis rate, serum creatinine level, and eGFR level) of the non-dialysis-dependent group was significantly better than that of the dialysis-dependent group (*p* < 0.001). The incidence of oliguria/anuria at admission was also remarkably higher in the dialysis dependent group (*p* = 0.003). Hemoglobin level was significantly higher in the non-dialysis dependent group (*p* = 0.017) (Table 2). Of the 53 patients who progressed to KFRT, 24 (45.3%) underwent renal biopsy. The patients with sclerotic disease (five of five; 100.0%) had the highest proportion those progressing to KFRT followed by those classified as crescentic (15 of 22, 68.2%), mixed (two of five, 40.0%), and focal (two of six, 33.3%).

**Table 2.** Clinical characteristics for different renal outcomes.

Variables	Non-dialysis dependent group (*n* = 23)	Dialysis dependent group (*n* = 53)	*p*
Male (*n*, %)	8 (34.8%)	26 (49.1%)	0.250
Age (years)	61.0 (39.0, 68.0)	60.0 (45.0, 70.5)	0.803
Duration of renal disease (months)	1.0 (0.5, 3.0)	1.0 (0.5, 2.0)	0.443
Oliguria/anuria (*n*, %)	1 (4.3%)	20 (37.7%)	0.003
Macroscopic hematuria (*n*, %)	11 (47.8%)	18 (34.0%)	0.253
Initial KRT (*n*, %)	8 (34.8%)	47 (88.7%)	<0.001
Anti-GBM antibody (RU/mL)	50.1 (34.6,112.7)	158.5 (115.5, 198.9)	<0.001
MPO-ANCA positivity (*n*, %)	5 (21.7%)	10 (18.9%)	0.762
Serum creatinine (μmol/L)	333.3 (119.9, 555.8)	781.3 (569.7, 956.3)	<0.001
eGFR (mL/min/1.73 m^2^)	15.2 (8.5, 40.1)	5.2 (4.1, 7.7)	<0.001
CRP (mg/L)	13.8 (3.4, 53.8)	47.4 (9.4, 128.5)	0.057
RF (IU/mL)	10.1 (9.6, 11.2)	10.8 (9.7, 21.4)	0.485
Serum albumin (g/L)	29.1 (25.7, 34.2)	27.5 (23.9, 32.5)	0.400
Hemoglobin (g/L)	88.0 (82.0, 115.0)	83.0 (70.0, 91.5)	0.017
Urine red blood cell (/μL)	593.0 (119.7, 1947.9)	1362.1 (386.5, 2407.0)	0.209
Urine protein (g/24 h)	1.32 (0.64, 5.01)	1.17 (0.39, 2.72)	0.175
Lung involvement (*n*, %)	7 (30.4%)	15 (28.3%)	0.851

Anti-GBM: anti-glomerular basement membrane; MPO-ANCA: myeloperoxidase-anti-neutrophil cytoplasmic antibody; eGFR: estimated glomerular filtration rate; KRT: kidney replacement therapy; CRP: C-reactive protein; RF: rheumatoid factors.

Oliguria was defined as urinary output of <400 mL/24 h while anuria was defined as urinary output of <100 mL/24 h. The Hospital Reference Laboratory normal range for anti-GBM antibody was 0–20 RU/mL; CRP was 0–8 mg/L; RF was 0–20 IU/mL; and urine red blood cell was 0–17/μL.

**Figure 1.**
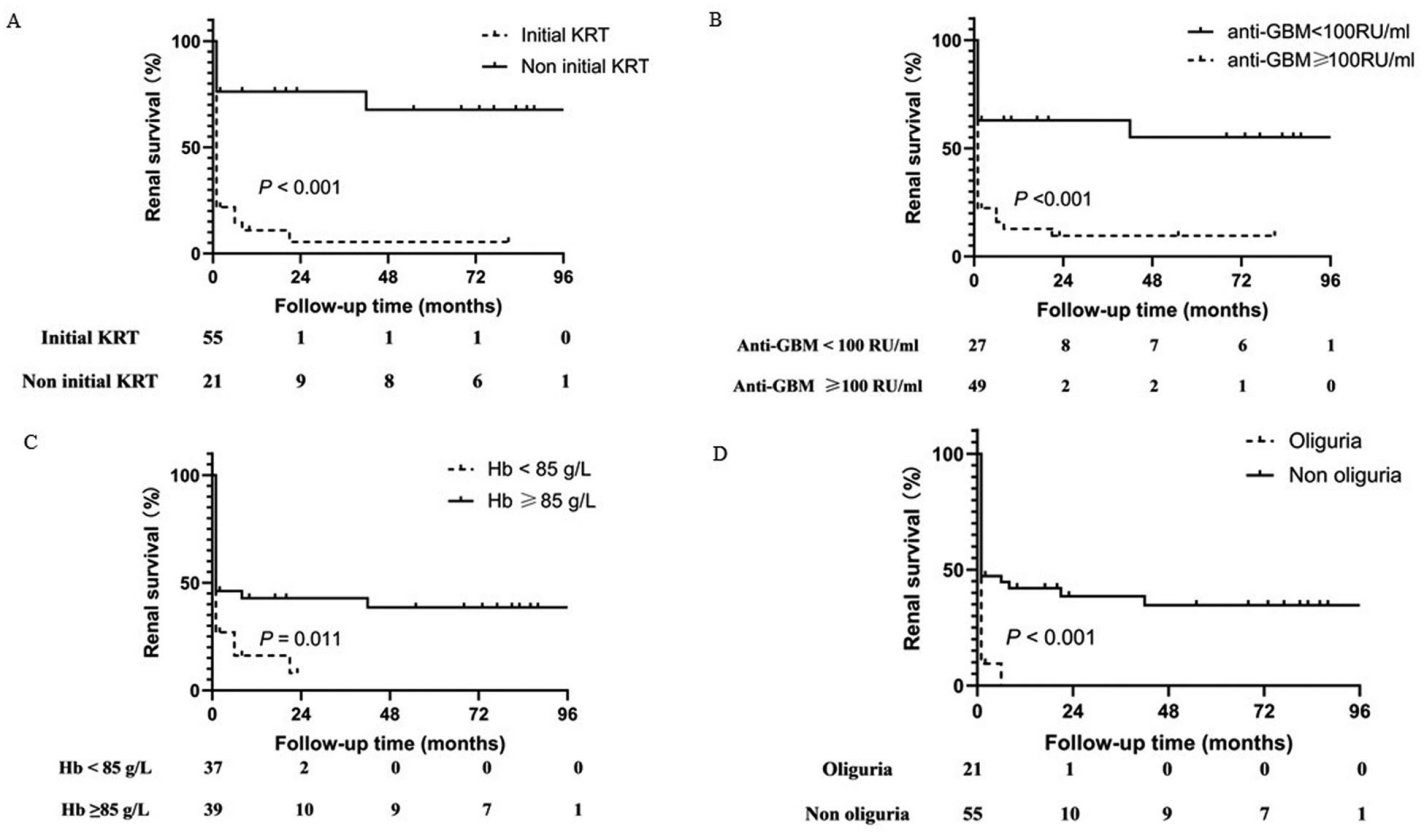
Kaplan–Meier’s renal survival curve for anti-GBM-GN and different risk factors. (A) Initial KRT, (B) anti-glomerular basement membrane (anti-GBM) level, (C) hemoglobin (Hb) level, and (D) oliguria.

Kaplan–Meier’s survival analysis indicated that patients with higher anti-GBM antibody levels (≥100 RU/mL, HR = 2.41, 95% CI = 1.38–4.18, *p<* 0.001), lower hemoglobin levels (<85 g/L, HR = 1.55, 95% CI = 0.90–2.66, *p=* 0.011), poorer initial renal function status (including initial KRT, serum creatinine, and eGFR) (*p* < 0.001) and oliguria (HR = 1.80, 95% CI = 0.97–3.34, *p* < 0.001) were more likely to progress to KFRT (Figure 1, Supplementary Figure 1). Cox regression analyses revealed that initial KRT (HR = 3.48, 95% CI = 1.22–9.97, *p* = 0.020) was a significant risk factor for renal survival (Table 3).

**Table 3.** Potential prognostic factors for kidney outcome by univariate and multivariate Cox’s regression analyses.

Variables	Univariable analysis			Multivariable analysis		
	HR	95% CI	*p*	HR	95% CI	*p*
Oliguria/anuria	1.84	1.04–3.26	0.036	1.18	0.64–2.16	0.593
Sex	1.26	0.74–2.17	0.397			
Age, ≥60 years	1.04	0.60–1.78	0.899			
Initial KRT	4.52	1.81–11.26	0.001	3.48	1.22–9.97	0.020
Serum albumin, <30 g/L	1.21	0.69–2.12	0.508			
MPO-ANCA	1.05	0.52–2.10	0.897			
Serum creatinine, ≥600 μmol/L	2.89	1.53–5.46	0.001			
eGFR	0.94	0.90–0.99	0.009			
Hemoglobin, <85 g/L	0.63	0.36–1.11	0.108			
Macroscopic hematuria	0.81	0.46–1.43	0.473			
Anti-GBM, ≥100 RU/mL	2.51	1.27–4.96	0.008	1.47	0.65–3.35	0.358
Lung involvement	0.91	0.50–1.66	0.754	0.74	0.39–1.41	0.366

MPO-ANCA: myeloperoxidase-anti-neutrophil cytoplasmic antibody; eGFR: estimated glomerular filtration rate; KRT: kidney replacement therapy; anti-GBM: anti-glomerular basement membrane.

### Risk factors affecting the survival of anti-GBM-GN patients

At the end of the follow up, 49 (64.5%) of 76 patients survived. Among the 27 patients who did not survive, seven died of severe secondary infection, which was the main cause of death; followed by five from anti-GBM disease activity, five from dialysis related complications, five from cancer (one cases of liver cancer, one cases of lung cancer, one cases of bladder cancer, and one cases of multiple myeloma). The cause of death of the remaining five non-surviving patients was unknown. Characteristics among groups of survivors and nonsurvivors were compared, as shown in Table 4.

**Table 4.** Clinical characteristics among survivors and non-survivors.

Variables	Survival group (*n* = 49)	Non-survival group (*n* = 27)	*p*
Male (*n*, %)	20 (40.8%)	14 (51.9%)	0.354
Age (years)	54.0 (38.5, 65.5)	69.0 (60.0, 82.0)	<0.001
Duration of renal disease (months)	1.0 (0.5, 2.5)	1.0 (0.5, 2.0)	0.991
Oliguria/anuria (*n*, %)	12 (24.5%)	9 (33.3%)	0.409
Macroscopic hematuria (*n*, %)	17 (34.7%)	12 (44.4%)	0.402
Initial KRT (*n*, %)	32 (65.3%)	23 (85.2%)	0.064
Anti-GBM antibody (RU/mL)	125.6 (45.4,192.1)	163.2 (56.8, 188.0)	0.345
MPO-ANCA positivity (*n*, %)	9 (18.4%)	6 (22.2%)	0.686
Serum creatinine (μmol/L)	582.6 (309.2, 852.6)	707.9 (398.4, 873.8)	0.252
eGFR (mL/min/1.73 m^2^)	7.2 (4.8, 16.1)	5.3 (4.3, 10.2)	0.102
CRP (mg/L)	18.7 (3.4, 114.8)	49.4 (15.4, 112.0)	0.204
RF (IU/mL)	10.8 (9.5, 11.4)	10.4 (10.0, 11.5)	0.492
Serum albumin (g/L)	29.1 ± 7.0	28.7 ± 4.8	0.810
Hemoglobin (g/L)	85.0 (78.0, 106.0)	81.0 (72.0, 89.0)	0.105
Urine red blood cell (/μL)	917.9 (264.1, 2029.3)	829.0 (313.7, 3950.2)	0.650
Urine protein (g/24 h)	1.24 (0.50, 3.94)	1.26 (0.37, 3.36)	0.457
Lung involvement (*n*, %)	13(26.5%)	9 (33.3%)	0.531

MPO-ANCA: myeloperoxidase-anti-neutrophil cytoplasmic antibody; eGFR: estimated glomerular filtration rate; KRT: kidney replacement therapy; anti-GBM: anti-glomerular basement membrane; CRP: C-reactive protein; RFs: rheumatoid factors.

Oliguria was defined as urinary output of <400 mL/24 h while anuria was defined as urinary output of <100 mL/24 h. The Hospital Reference Laboratory normal range for anti-GBM antibody was 0–20 RU/mL; CRP was 0–8 mg/L; RF was 0–20 IU/mL; and urine red blood cell was 0–17/μL.

**Figure 2.**
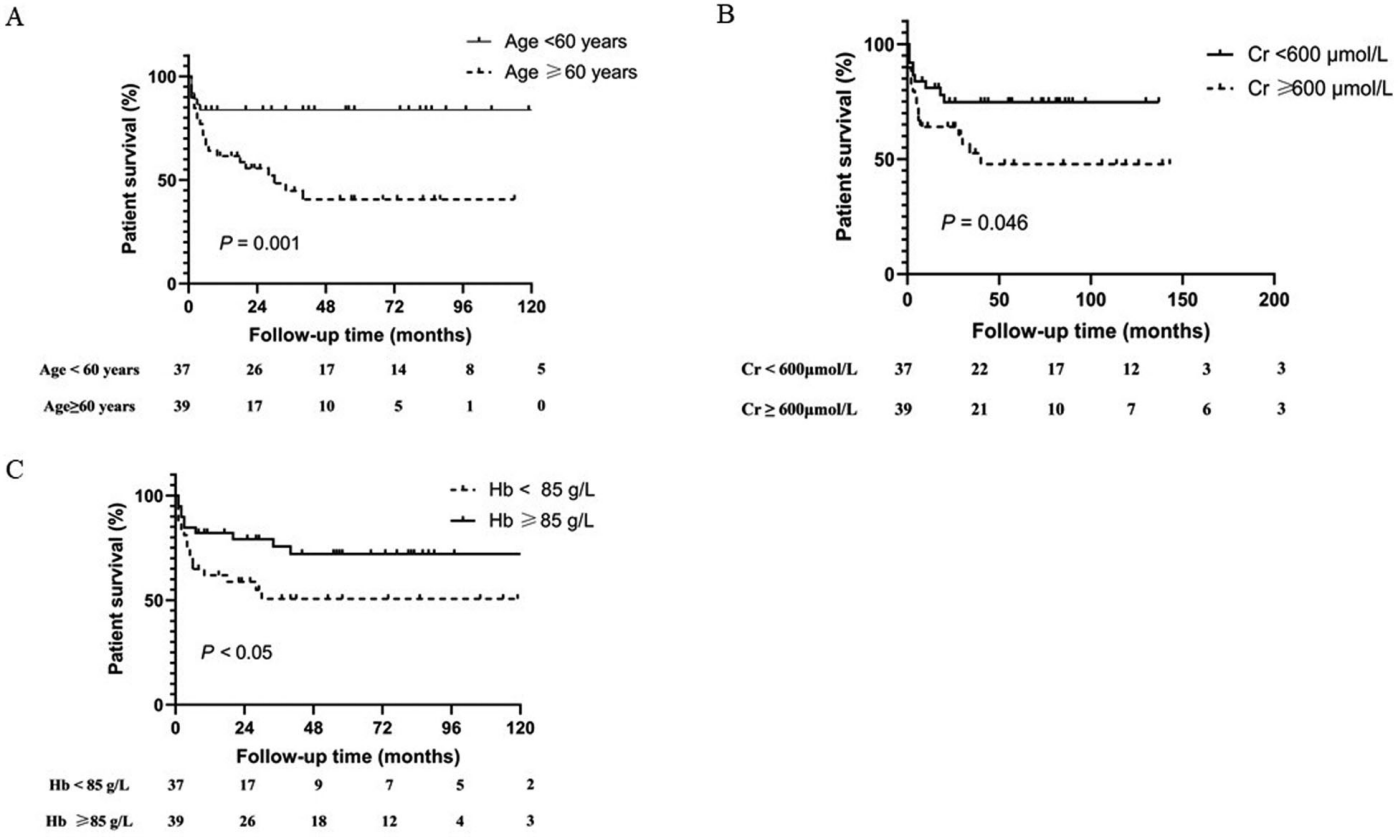
Kaplan–Meier’s patient survival curve for anti-GBM-GN with different risk factors. (A) Age, (B) serum creatinine (Cr) level, and (C) hemoglobin (Hb) level.

Kaplan–Meier’s survival analysis indicated that younger age (<60 years, HR = 0.26, 95% CI = 0.12–0.55, *p* = 0.001), higher hemoglobin levels (≥85 g/L, HR = 0.47, 95% CI = 0.22–1.01, *p* = 0.049), and lower serum creatine levels (<600 μmol/L, HR = 0.46, 95% CI = 0.22–0.98, *p* = 0.046) were associated with better patient survival (Figure 2). After adjusting for clinical baseline parameters, including hemoglobin, creatinine, and treatment regimens, Cox regression analyses showed that the older age (≥60 years, HR = 4.13, 95% CI = 1.65–10.38, *p* = 0.003) and the initial KRT (HR = 2.87, 95% CI = 1.01–8.14, *p* = 0.047) were significant risk factors affecting patient survival (Table 5).

**Table 5.** Potential prognostic factors for patient outcome by univariate and multivariate Cox regression analyses.

Variables	Univariable analysis			Multivariable analysis		
	HR	95% CI	*p*	HR	95% CI	*p*
Age, ≥60 years	3.91	1.57–9.72	0.003	4.13	1.65–10.38	0.003
Sex	1.46	0.68–3.11	0.328			
Anti-GBM, ≥100 RU/mL	1.70	0.72–4.02	0.228			
MPO-ANCA	1.23	0.49–3.05	0.658			
Creatine, ≥600 μmol/L	2.19	0.98–4.88	0.056			
eGFR	0.99	0.97–1.01	0.390			
Initial KRT	2.47	0.85–7.16	0.096	2.87	1.01–8.14	0.047
Macroscopic hematuria	1.35	0.63–2.90	0.434			
Oliguria/anuria	1.43	0.64–3.18	0.384			
Hemoglobin, <85 g/L	0.47	0.21–1.03	0.059	1.54	0.62–3.79	0.349
Lung involvement	1.26	0.57–2.81	0.567	1.34	0.58–3.06	0.491

MPO-ANCA: myeloperoxidase-anti-neutrophil cytoplasmic antibody; eGFR: estimated glomerular filtration rate; KRT: kidney replacement therapy; anti-GBM: anti-glomerular basement membrane.

## Discussion

Anti-GBM disease is a rare disease, with an estimated incidence ranging from 0.5 to 1.6 per million per year [2]. Similar to the above study, the incidence in Chinese population is 0.6 per million per year [19]. Anti-GBM-GN accounts for 10–15% of all crescentic GN cases in large-scale biopsy series, although it seems to be a rare cause leading to KFRT [20,21]. The severity and rapid progression of the disease require an early diagnosis for the sake of quick initiation of plasma exchange and immunosuppressive therapy. There are still unmet needs to ascertain prognostic factors ahead of complications to target patients demanding active therapy. As one of the larger sample size studies on anti-GBM-GN, we studied 76 patients with anti-GBM-GN hospitalized between 2010 and 2021. The results indicated that initial KRT was a significant risk factor for renal survival, while patient survival was influenced by advanced age and initial KRT. The study also demonstrated that renal recovery or partial recovery from dialysis was rare among patients who were dialysis-dependent entry. At the same time, we found that the titer of anti-GBM antibodies did not affect the renal function or survival of patients.

In this study, data of anti-GBM-GN patients were analyzed based on ANCA status. 19.7% of patients had positive ANCA, and these patients were older. Consistently with previous studies, we also reported a relatively high frequency of anti-GBM-GN patients with positive MPO-ANCA [7,22]. Olson et al. found that ANCA-induced glomerular inflammation may trigger the development of anti-GBM response, possibly via modifying or exposing generally sequestered disease epitopes in GBM, as it has been found that ANCA may be usually detected earlier than the onset of anti-GBM disease [23]. Our double positive patients had a higher incidence of acute kidney injury at presentation, so they also had a relatively high rate of initial KRT. In a large multicenter cohort study, McAdoo et al. showed that double-positive patients had severe lung and kidney injury at presentation, requiring plasma exchange and aggressive immunosuppressive therapy. During long-term follow-up, they relapsed at a frequency similar to a parallel cohort of ANCA-associated vasculitis (AAV) patients, suggesting that the patients had a truly hybrid disease phenotype, needing early aggressive treatment for anti-GBM disease, and detailed long-term follow-up and consideration for maintenance immunosuppression for AAV [7].

Similar to some previous studies of anti-GBM disease, a multicenter study of 119 patients performed by Marques et al. found that renal outcome was significantly poorer in patients with older age at diagnosis, female gender, and high level of serum creatinine at presentation [15]. In a 15-year retrospective study of 48 patients with biopsy-proven renal involvement of anti-GBM disease, oliguria, high serum creatinine, severe glomerulosclerosis, and severe tubular atrophy and interstitial fibrosis were proved to be linked with poor renal survival [24]. In another Chinese study with 221 patients, after analyzing data from 221 consecutive patients, Cui et al. demonstrated that the serum creatinine at presentation was an independent predictor for renal failure [16]. van Daalen et al. also confirmed that dialysis dependency at presentation was predictive of poor kidney outcomes. In terms of renal histopathology, large extent of interstitial infiltrate and low percentage of normal glomeruli were related to poor kidney outcome in anti-GBM-GN [14]. In accordance with most current studies, the results of the present study also found that the initial KRT was predictive of renal survival. Anemia is common along with anti GBM disease. Previous studies reported that the incidence of anemia was 83.3% and the average hemoglobin concentration was about 90 g/L [15,24]. Our previous study found that low hemoglobin level was connected to a greater KFRT risk in patients with MPO-ANCA associated glomerulonephritis [25]. However, the present study did not find that anemia was a related factor in the progression of KFRT.

The present study revealed that older age and the initial KRT were significant predictors for survival of patients with anti-GBM disease. As reported in previous studies, age was acknowledged as an important risk factor of the survival of patients with anti-GBM disease. However, our study found that initial KRT was also a risk factor of survival of patients, which rarely mentioned in previous studies. The mortality rate of anti-GBM disease was used to be very high, mainly attributing to pulmonary hemorrhage or KFRT [26]. Recent treatment options including plasma exchange, GC, and cyclophosphamide significantly improved the prognosis of patients. Zahir et al. found that advanced age, high serum creatinine and high anti-GBM titers were associated with poor patient survival [24]. Advanced age, as an independent predictor of poor patient survival, has been also proved by others [13,15]. In contrast, most studies found no relationship between initial KRT and patient outcomes. A national cohort study from France analyzed 122 patients with Goodpasture’s syndrome and found that in addition to age, another predictive factor affecting the outcomes of patients was the number of plasma exchanges [12]. These findings emphasized the significance of early diagnosis to improve prognosis of patients with anti-GBM disease. In a Chinese retrospective study of older patients presenting with anti-GBM disease, Cui et al. demonstrated that kidney damage on diagnosis was an independent predictor for survival of elderly patients [11]. In another large cohort of 221 Chinese anti-GBM patients, age, renal involvement, ANCA positivity, plasma exchange, and steroid use were considered to be associated with the overall survival of patients [16]. The above studies partially demonstrated that the initial KRT was related to the outcomes of anti-GBM patients. Consistent with the present study, another multicenter study involving 123 patients with anti-GBM-GN found that dialysis independency at presentation, high percentage of normal glomeruli, and low extent of interstitial infiltrate were related to a favorable outcome [14].

This study also has some limitations. First, this was a single center study with a retrospective design, and the sample size is relatively small, thus the results were liable to possible selection bias. Second, renal biopsy was performed for only 38 (50.0%) of the 76 patients due to poor renal function at the time of diagnosis requiring initial KRT or the potential risk of complications. Therefore, the renal and patient outcomes may not be fully analyzed from the perspective of renal histopathology. Third, in terms of treatment, especially the proportion of patients receiving plasma exchange was relatively low, which may also have a certain impact on the analysis of renal and patient outcomes. Fourth, GC plus CTX treatment was administered to the majority of patients in this study, so the choice of treatment may lead to biased results.

As one of the few studies with relatively large sample size on anti-GBM-GN, the results of the present study confirmed that initial KRT is associated with renal outcomes. In terms of patient outcomes, age and the initial KRT are confirmed as predictors in this study.

## Supplementary Material

Supplemental Material

Supplemental Material

Supplemental Material
