# Supplementary material for: Clinicopathological characteristics and outcome predictors of anti-glomerular basement membrane glomerulonephritis
Source: Ren Fail. 2022 Nov 21;44(1):2037–45. doi: 10.1080/0886022X.2022.2147673 (PMC9683053; doi:10.1080/0886022X.2022.2147673)
Supplement: Supplemental Material [file IRNF_A_2147673_SM0004.pdf]

Supplementary Table 2 Clinical characteristics of patients with anti-GBM disease by ANCA positivity

Variables	Anti-GBM alone (n=61)	Double positive (n=15)	*p*
Male (n, %)	27 (44.3%)	7 (46.7%)	0.867
Age (years)	56 (41.5, 67.5)	68.0 (61.0, 74.0)	0.030
Duration of renal disease (months)	1.0 (0.5, 2.0)	1.0 (0.5, 2.0)	0.967
Oliguria/anuria (n, %)	18 (29.5%)	3 (20.0%)	0.538
Macroscopic hematuria (n, %)	24 (39.3%)	5 (33.3%)	0.668
Initial RRT (n, %)	42 (68.9%)	13 (86.7%)	0.212
Serum creatinine (μmol/L)	589.1 (314.0, 875.5)	698.8 (429.9, 814.7)	0.643
eGFR (mL/min/1.73m^2)	6.7 (4.4, 15.6)	5.4 (4.4, 10.2)	0.407
CRP (mg/L)	34.4 (6.4, 120.3)	19.7 (4.4, 83.6)	0.557
RF (IU/mL)	10.7 (9.7, 11.4)	10.8 (9.5, 21.2)	0.840
Serum Albumin (g/L)	28.3 (23.5, 32.1)	28.5 (25.9, 34.2)	0.588
Hemoglobin (g/L)	85.0 (74.5, 103.5)	83.0 (74.0, 86.0)	0.354
Urine red blood cell (/μL)	942.0 (324.8, 2371.9)	829.0 (146.7, 1871.0)	0.705
urine protein (g/24h)	1.32 (0.50, 3.98)	0.68 (0.44, 2.26)	0.267
Lung involvement	18 (29.5%)	4 (26.7%)	1.000

(n, %)

eGFR, estimated glomerular filtration rate; RRT, renal replacement therapy; CRP, C-reactive protein; RF, rheumatoid factors.

Among 15 double positive patients, the immunofluorescence of the 5 patients receiving renal biopsy mainly showed that IgG deposited linearly along GBM. Electron microscopy showed that there was no electronic dense deposit.

Oliguria was defined as urinary output of <400 mL/24h while anuria was defined as urinary output of <100 mL/24h

The Hospital Reference Laboratory normal range for anti-GBM antibody was 0-20 RU/ml; CRP was 0-8 mg/L; RF was 0–20 IU/ml; and urine red blood cell was 0-17/μL.
