# Supplementary material for: Clinicopathological characteristics and outcome predictors of anti-glomerular basement membrane glomerulonephritis
Source: Ren Fail. 2022 Nov 21;44(1):2037–45. doi: 10.1080/0886022X.2022.2147673 (PMC9683053; doi:10.1080/0886022X.2022.2147673)
Supplement: Supplemental Material [file IRNF_A_2147673_SM9963.pdf]

Supplementary Table 1 Pathological features of different histopathological types of anti-GBM disease in 38 patients undergoing renal biopsy

	Focal (n=6)	Mixed (n=5)	Crescentic (n=22)	Sclerotic (n=5)	*p*
Total glomeruli	18.5 (15.0, 30.5)	13.0 (8.5, 24.0)	19.0 (15.0, 26.0)	12.0 (10.5, 21.0)	0.355
Normal glomeruli (%)	71.4 (63.2, 89.6)	17.8 (3.8, 33.9)	5.8 (0.0, 11.7)[a]	0.0 (0.0, 15.3)[a]	**< 0.001**
Global sclerotic glomeruli (%)	8.5 (0, 13.0)	20.0 (0, 35.0)	0.0 (0, 6.9)	66.7 (55.8, 84.3)[a,c]	**< 0.001**
Cellular crescents (%)	9.2 ± 10.9	27.4 ± 16.6	76.7 ± 15.6[a,b]	11.2 ± 12.7[c]	**< 0.001**
Acute tubulointerstitial lesions score	1.5 (1.0, 2.3)	3.0 (2.0, 3.0)	3.0 (3.0, 3.0)[a]	3.0 (2.5, 3.0)[a]	**< 0.001**
Chronic tubulointerstitial lesions score	1.0 (1.0, 1.0)	2.0 (1.0, 2.0)	1.0 (1.0, 1.0)	2.0 (1.5, 2.0)	**0.038**
Tubular atrophy and Interstitial fibrosis (%)	25.0 (13.8, 32.5)	30.0 (22.5, 35.0)	22.5 (20.0, 25.0)	50.0 (40.0, 50.0)[a,c]	**0.002**

[a]Focal versus mixed, crescentic, and sclerotic ($p < 0.05$).

[b]Mixed versus crescentic and sclerotic ($p < 0.05$).

[c]Crescentic versus sclerotic ($p < 0.05$).
