# Supplementary figures and images for: Clinicopathological characteristics and outcome predictors of anti-glomerular basement membrane glomerulonephritis
Source: Ren Fail. 2022 Nov 21;44(1):2037–45. doi: 10.1080/0886022X.2022.2147673 (PMC9683053; doi:10.1080/0886022X.2022.2147673)

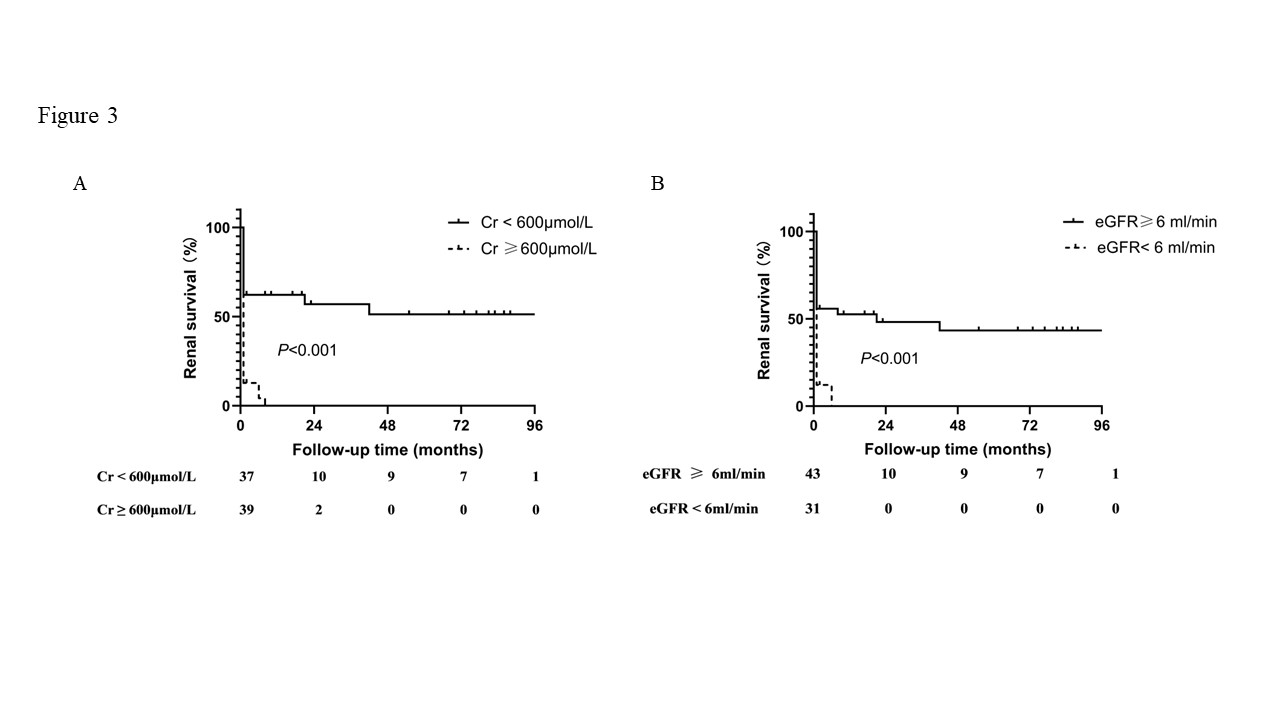

Supplement: Supplemental Material [file IRNF_A_2147673_SM9953.jpg]
